# Saltatory formation, sliding and dissolution of ER–PM junctions in migrating cancer cells

**DOI:** 10.1042/BJ20121864

**Published:** 2013-03-14

**Authors:** Hayley Dingsdale, Emmanuel Okeke, Muhammad Awais, Lee Haynes, David N. Criddle, Robert Sutton, Alexei V. Tepikin

**Affiliations:** *Department of Cellular and Molecular Physiology, The University of Liverpool, Crown Street, Liverpool L69 3BX, U.K.; †NIHR (National Institute of Health Research) Liverpool Pancreas Biomedical Research Unit, The University of Liverpool, Crown Street, Liverpool L69 3BX, U.K.

**Keywords:** actin, endoplasmic reticulum–plasma membrane junctions (ER–PM junctions), PANC-1 cells, store-operated Ca^2+^ entry (SOCE), vinculin, CFP, cyan fluorescent protein, CPA, cyclopiazonic acid, DMEM, Dulbecco's modified Eagle's medium, ER, endoplasmic reticulum, FBS, fetal bovine serum, FKBP, FK506-binding protein, FRB, fragment of mammalian target of rapamycin that binds FKBP12, GFP, green fluorescent protein, mRFP, monomeric red fluorescent protein, Orai, Ca^2+^ release-activated Ca^2+^ channel protein, PM, plasma membrane, RT, room temperature, SOCE, store-operated Ca^2+^ entry, STIM, stromal interaction molecule, YFP, yellow fluorescent protein

## Abstract

We demonstrated three novel forms of dynamic behaviour of junctions between the ER (endoplasmic reticulum) and the PM (plasma membrane) in migrating cancer cells: saltatory formation, long-distance sliding and dissolution. The individual ER–PM junctions formed near the leading edge of migrating cells (usually within 0.5 μm of polymerized actin and close to focal adhesions) and appeared suddenly without sliding from the interior of the cell. The long distance sliding and dissolution of ER–PM junctions accompanied the tail withdrawal.

## INTRODUCTION

The recent discovery of the mechanism of SOCE (store-operated Ca^2+^ entry) propelled the junctions between the ER (endoplasmic reticulum) and the PM (plasma membrane) into the limelight of the cell signalling research field (reviewed in [1]). SOCE is initiated by a decrease in the Ca^2+^ concentration in the ER ([Ca^2+^]_ER_) [2]; the decrease is detected by STIM (stromal interaction molecule) proteins, which form oligomers, translocate to ER–PM junctions and then activate Orai (Ca^2+^ release-activated Ca^2+^ channel protein) channels in the PM [3–7]. Crucially, direct contact between STIM (transmembrane proteins in the ER) and Orai (transmembrane proteins in the PM) proteins is necessary to activate SOCE channels [5,7]. This is only possible in structures where the ER membrane and PM are very close to one another, i.e. in ER–PM junctions. The distance between the membranes in such junctions is less than 25 nm [8–11] and ribosomes are specifically excluded [8,11]. Previous studies reported both stationary ER–PM junctions [8] and junctions which rapidly form as a result of the translocation of ER strands towards the PM [10]. ER–PM junctions are not only structural platforms for Ca^2+^ signalling; they also play an integral role in the initiation of cAMP responses [12,13]. A number of recent studies indicated the importance of both Ca^2+^ and cAMP signals in cell migration ([14–18] and reviewed in [19,20]). We therefore decided to characterize the localization and dynamics of the ER–PM junctions in migrating cancer cells.

## MATERIALS AND METHODS

### Cells, reagents and constructs

PANC-1 cells obtained from the A.T.C.C. (ATCC number CRL-1469) were cultured in DMEM (Dulbecco's modified Eagle's medium) supplemented with 10% FBS (fetal bovine serum), 100 units/ml penicillin, 100 μg/ml streptomycin and 292 μg/ml glutamine. YFP (yellow fluorescent protein)–STIM1 and mCherry–Orai1 [both with a CMV (cytomegalovirus) promoter] were described previously [21]; as expected YFP–STIM1 translocates to puncta (revealing ER–PM junctions) and co-clusters with mCherry–Orai1 in cells treated with CPA (cyclopiazonic acid; *n*=66, results not shown; here and below *n* indicates the number of cells unless indicated otherwise). YFP–STIM1 [with a TK (thymidine kinase) promoter] [9] was a gift from Dr T. Balla (National Institute of Child Health and Human Development, Bethesda, MD, U.S.A.). YFP–STIM1(D76A) was from Addgene (plasmid 18859; [4]). The YFP–STIM1(NN) mutant was constructed using standard molecular biology procedures and based on the construct described previously [22]. To reveal the ER–PM junctions independently of ER Ca^2+^ store depletion, STIM1 translocation and STIM1–Orai1 interaction, we utilized rapamycin-inducible linkers developed by Dr T. Balla [9]. To form such linkers one of the interacting proteins [LL–FKBP (FK506-binding protein where LL indicates that a longer helical linker was used)–mRFP (monomeric red fluorescent protein)] was targeted to the PM and another [CFP (cyan fluorescent protein)–FRB (fragment of mammalian target of rapamycin that binds FKBP12)–LL] to the cytosolic surface of the ER. Targeting of LL–FKBP–mRFP to the PM was achieved by attaching the N-terminal palmitoylation/myristoylation signal of the Lyn protein; targeting of the CFP–FRB–LL protein to the ER membrane was attained using the C-terminal localization sequence of Sac1 phosphatase [9]. These rapamycin-inducible constructs with longer helical linkers (specifically PM-targeted LL–FKBP–mRFP and ER-targeted CFP–FRB–LL) [9] were gifts from Dr T. Balla. LifeAct-TagRFP was from Ibidi. The anti-β-actin (clone AC-15), polyclonal anti-calnexin and anti-vinculin (clone hVIN-1) antibodies were purchased from Sigma–Aldrich. The anti-GFP (green fluorescent protein) antibody and Alexa Fluor® 647-conjugated phalloidin were from Invitrogen. Alexa Fluor®-conjugated secondary antibodies were from Invitrogen. CPA was from Tocris and rapamycin was from Calbiochem. SYTOX® Orange and Hoechst 33342 were from Invitrogen.

### Confocal microscopy

For imaging of migrating PANC-1 cells, the cells were seeded into 35 mm glass-bottom dishes and transfected 24 h later using Promofectin (Promokine) as per the manufacturer's instructions. Immediately prior to imaging, the medium was changed to a solution based on DMEM (the basal serum-free and Ca^2+^-free medium from Invitrogen) to which CaCl_2_ was added to attain the required Ca^2+^ concentration (1 mM Ca^2+^ in the majority of the experiments), supplemented with 15 μM CPA, 10% (v/v) FBS, 100 units/ml penicillin, 100 μg/ml streptomycin and 292 μg/ml glutamine. Overnight (approximately 20 h) live imaging of cells was performed using a Zeiss 710 laser-scanning confocal microscope, with cells kept at 37°C and 5% CO_2_. Under the conditions of our experiments 15 μM CPA did not induce strong cellular toxicity: even after 21 h in CPA-containing medium the majority of cells (106 out of 111) were alive as assessed using a combination of SYTOX® Orange probe to reveal cells with compromized plasma membranes and Hoechst 33342 to stain all of the cells' nuclei. In the control experiments (without CPA) 114 out of 117 cells were alive after 21 h of incubation. Imaging of fixed cells (and short-term imaging of live cells) was carried out using a Leica TSC SP2 AOBS confocal microscope (Leica Microsystems).

### Immunofluorescence

Cells were seeded either on to coverslips or into 35 mm glass-bottom dishes (Mattek). Fixation was performed using either 100% methanol for 10 min at −20°C or 4% (v/v; diluted in PBS) PFA for 30 min at RT (room temperature; 19–21°C). The cells were then washed three times with PBS. Where PFA fixation was used, the cells were subsequently permeabilized using 0.1% Triton X-100 (v/v; diluted in PBS) for 5 min at RT, before an additional three PBS washes. Blocking was carried out for 1 h at RT in PBS containing 10% (v/v) goat serum and 1% (w/v) BSA. Primary antibodies were added at the following dilutions: anti-GFP, 1:200; anti-β-actin, 1:400; anti-vinculin, 1:200; and anti-calnexin, 1:100 and phalloidin was added during the secondary antibody stage at 1:50 dilution. The antibodies were added in a PBS solution containing 5% (v/v) goat serum and 0.1% acetylated BSA for 1 h at RT, before three PBS washes and the addition of secondary antibodies at 1:500–1:1000 dilution in PBS for 20 min at RT. Cells were then washed three times in PBS before mounting on to microscope slides (Thermo Scientific) using ProLong Gold (Invitrogen).

### Image analysis

Image acquisition and initial analysis was carried out using either Zeiss Zen or Leica LAS software; further analysis was performed using ImageJ software (http://rsbweb.nih.gov/ij/). Only linear adjustments of brightness and contrast were used.

The ‘mask’ images used for illustrating the co-localization of linker components (images labelled ER&PM Linkers) were created using the RG2B Co-localization ImageJ plugin (developed by C.P. Mauer, Northwestern, Evanston, IL, U.S.A.). The image showing just the regions of co-localization between the two linker constructs was created by using this plugin and adjusting threshold values until the resulting image matched the co-localization seen when the raw images were overlaid (excluding non-co-localizing fluorescence from either channel).

## RESULTS AND DISCUSSION

**Figure 1:**
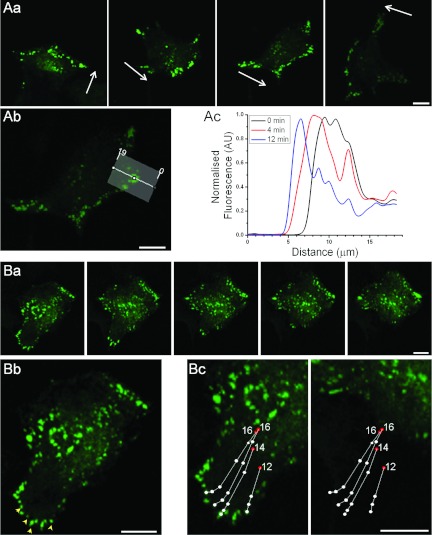
ER–PM junctions in migrating PANC-1 cells In this experiment YFP–STIM1-transfected PANC-1 cells were treated with CPA to reveal the positioning of ER–PM junctions. Cells were imaged overnight. In this and other Figures the confocal section closest to the coverslip is shown. (**A**) Formation of puncta at the front of a migrating cell. (**Aa**) Images of a migrating cell; the time sequence is from left- to right-hand side and the arrows represent the direction of migration. The dynamics of the STIM1 puncta in this cell is shown in a Supplementary Movie S1 (at http://www.biochemj.org/bj/451/bj4510025add.htm). (**Ab**) Shows the region at the front of the cell selected for analyses, the distribution of fluorescence in the region highlighted by the bar was plotted along the white line with indicated distances (in μm) in (**Ac**). (**Ac**) The distribution of fluorescence recorded in the region highlighted in (**Ab**) at different time points starting from the beginning of the analyses. (**B**) Long-distance sliding and dissolution of puncta during tail retraction (same cell as in **A**). (**Ba**) Image series depicting tail retraction events. Note the disappearance of puncta in the tail that occurs during the retraction. The time interval between the first and last image is 18 min. (**Bb**) Four puncta (yellow arrowheads) were tracked during the tail retraction in (**Ba**) and their movement plotted in (**Bc**). (**Bc**) Traces showing the sliding of puncta during the tail retraction were superimposed on the fragment of the cell image at the beginning (left-hand panel) and the end (right panel-hand; 18 min from the beginning) of the tail retraction. The dots represent positions of the STIM1 puncta at 0, 8, 10, 12, 14 and 16 min (the red dots at the end of the lines indicate the last recorded positions of the puncta; the numbers by the red dots indicate the time of the disappearance of puncta in min from the beginning of the tail withdrawal). The scale bars in (**A**) and (**B**) represent 10 μm. The sliding and dissolution of the STIM1 puncta in this cell is also shown in a Supplementary Movie S2 (at http://www.biochemj.org/bj/451/bj4510025add.htm).

In the present study we used the pancreatic ductal adenocarcinoma cell line PANC-1 to investigate the dynamics of ER–PM junctions in migrating cells. The ability to migrate is essential for the metastatic phenotype of this and other types of cancers. It is important to note that the depletion of [Ca^2+^]_ER_ did not prevent migration of PANC-1 cells (Supplementary Figure S1 at http://www.biochemj.org/bj/451/bj4510025add.htm); the lack of inhibition (and actually some potentiation) was reported previously for another cell type [23]. The depletion of [Ca^2+^]_ER_ in YFP–STIM1-expressing cells allowed us therefore to reveal the localization and study the dynamics of ER–PM junctions in moving cells. We imaged migrating CPA-treated YFP–STIM1 cells using confocal microscopy and observed a prominent group of peripheral YFP–STIM1 puncta (i.e. ER–PM junctions) continuously forming close to the leading edge of the cell during migration (*n*=12, Figure 1A and Supplementary Movie S1 at http://www.biochemj.org/bj/451/bj4510025add.htm). It was also possible to observe some central and tail-located YFP–STIM1 puncta and, importantly, a region largely devoid of puncta just behind the leading edge of the cell (Figure 1A). A similar distribution of puncta was observed using the STIM1 EF-hand mutant (D76A) [4], which is concentrated in ER–PM junctions of cells with intact (i.e. not depleted) ER Ca^2+^ stores (Supplementary Figure S2 at http://www.biochemj.org/bj/451/bj4510025add.htm). Two other new forms of behaviour of YFP–STIM1 puncta are the sliding and dissolution that occur during the withdrawal of the tail of migrating cells. The sliding and dissolution are illustrated in Figure 1(B) (*n*=14) (note the movement and disappearance of the puncta during the shortening of the tail) and in Supplementary Movie S2 (at http://www.biochemj.org/bj/451/bj4510025add.htm). The results of the present study therefore suggest that ER–PM junctions are highly dynamic and can undergo rapid formation, sliding and dissolution, and that these processes are co-ordinated with cell migration.

**Figure 2:**
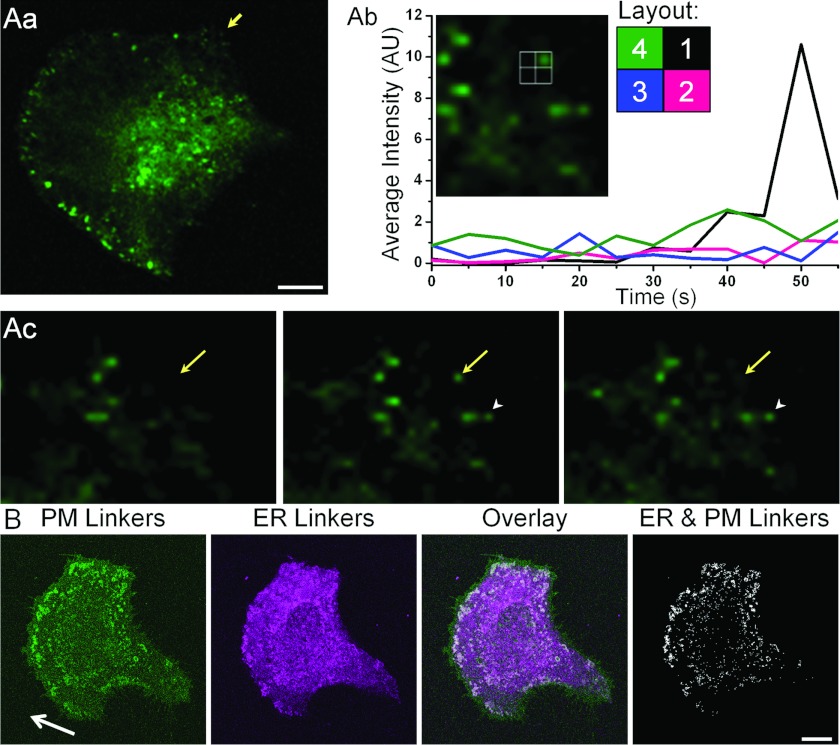
Saltatory formation and frontal positioning of ER–PM junctions (**A**) Saltatory formation of ER–PM junctions at the leading edge of migrating cells. YFP–STIM1-transfected PANC-1 cells were treated with CPA and imaged using a confocal microscope. (**Aa**) Image of a cell showing the area selected for analysis of puncta formation (indicated by an arrow). (**Ab**) Fragment of (**Aa**), with four regions of interest, one includes the newly formed punctum (region 1) and three (regions 2–4) include the neighbouring peripheral regions of the cell. The graph shows fluorescence intensity over time in each region; the colour of the traces corresponds to the colour of the regions according to the depicted layout. The sudden increase in fluorescence in region 1 reflects the punctum formation. (**Ac**) Images showing cell region before, during and after the punctum appearance, with yellow arrows highlighting the punctum [the same punctum as in (**Ab**)]. The white arrowhead shows another punctum which formed at approximately the same time, but that was still present at the end of recording. (**B**) Rapamycin-inducible ER–PM linkers reveal clustering of ER–PM junctions at the leading edge of migrating cells. PANC-1 cells transfected with PM-targeted LL–FKBP–mRFP and ER-targeted CFP–FRB–LL were imaged live to determine the direction of migration, and then treated with rapamycin to reveal the ER–PM junctions. In these experiments ER Ca^2+^ stores have not been depleted. Arrow shows direction of migration. Note co-clustering of ER and PM markers (white colour) at the leading edge of the migrating cell. The distribution of fluorescence before addition of rapamycin is shown in Supplementary Figure S5 (at http://www.biochemj.org/bj/451/bj4510025add.htm). The scale bars in (**A**) and (**B**) represent 10 μm. AU, arbitrary units.

The slow overnight imaging allowed us to determine the general trend of puncta dynamics near the leading edge of migrating cells. We next used faster imaging to investigate the dynamics of the formation of individual ER–PM junctions at the leading edge. We found that at the leading edge the YFP–STIM1 puncta are formed by a saltatory mechanism, i.e. they do not slide from the cell interior, but instead suddenly appear at the cell periphery (Figure 2A, *n*=27). The new puncta could then dissolve (see the punctum shown by yellow arrow on Figure 2Ac) or stabilize (see punctum shown by white arrowhead on Figure 2Ac). Another process that drives accumulation of STIM1 into ER–PM junctions is an increase in temperature [24]; under such conditions the depletion of the ER Ca^2+^ stores is not required to reveal the junctions. Saltatory formation of peripheral puncta was observed in live cells exposed to an increased temperature (*n*=25, Supplementary Figure S3 at http://www.biochemj.org/bj/451/bj4510025add.htm), demonstrating that this process can be revealed through different mechanisms of STIM1 accumulation in the junctions and that it does not require ER Ca^2+^ depletion. The potential role of microtubules in the saltatory formation of peripheral ER–PM junctions was investigated using the YFP–STIM1(NN) mutant that can no longer bind EB1 (end-binding protein 1) [22] and therefore does not associate with microtubules. We observed saltatory formation of puncta in cells expressing YFP–STIM1(NN) suggesting that this process is unlikely to be microtubule dependent (*n*=7, Supplementary Figure S4 at http://www.biochemj.org/bj/451/bj4510025add.htm). The results of all these experiments revealed and confirmed the saltatory formation of ER–PM junctions in the vicinity of the leading edge of migrating cells.

The localization of the junctions was further investigated using fluorescently labelled ER and PM proteins which bind to one another on the addition of rapamycin, but only at junctions [9]. This is another alternative mechanism to reveal ER–PM junctions and the co-localization of junctions revealed by such rapamycin-inducible linkers and STIM1 puncta has been reported previously ([9]; we also re-confirmed this in the present study, *n*=3, results not shown). The experiments with rapamycin-inducible linkers revealed an increased density of ER–PM junctions near the leading edge (*n*=27, Figure 2B and Supplementary Figure S5 at http://www.biochemj.org/bj/451/bj4510025add.htm) confirming the conclusion from the experiments with YFP–STIM1 regarding the preferential clustering of ER–PM junctions in this region.

**Figure 3:**
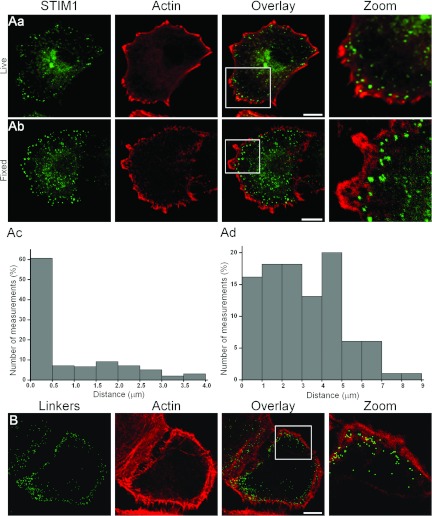
ER–PM junctions are located immediately behind actin-enriched regions at the leading edge of migrating cells (**A**) Relationship between STIM1 puncta and actin distribution in cells with depleted ER Ca^2+^ stores. (**Aa**) A live PANC-1 cell transfected with RFP-LifeAct (red) and YFP–STIM1 (green). Note the groups of puncta adjacent to regions of polymerized actin. (**Ab**) A store-depleted fixed YFP–STIM1-transfected PANC-1 cell immunostained for actin and YFP. (**Ac**) The distance between STIM1 puncta and the inside edge of actin (measurements were conducted on fixed cells). The histogram is based on 99 measurements of distances between puncta and the closest points on the inner actin edge (taken from nine cells). For each cell 3.6 μm of a randomly selected part of the inner actin edge and corresponding peripheral puncta (up to 4 μm in from the inside actin edge) were analysed. (**Ad**) The distance between STIM1 puncta and the outside edge of actin. The distances to the closest point on the outer edge of actin were measured from the same puncta as analysed in (**Ac**). (**B**) Relative distribution of linker-delineated junctions and actin in PANC-1 cells. In these experiments ER Ca^2+^ stores have not been depleted. PANC-1 cells transfected with rapamycin-inducible linkers were treated with rapamycin to reveal the ER–PM junctions. The green colour in the left-hand panel highlights the structures in which staining of the ER and PM markers is co-localized following the addition of rapamycin. Actin localization was revealed by staining with Alexa Fluor® 647-conjugated phalloidin. The scale bars in (**A**) and (**B**) represent 10 μm.

**Figure 4:**
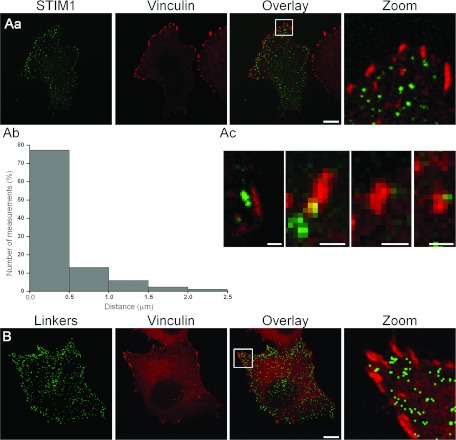
ER–PM junctions and focal adhesions (**A**) The relationship between the ER–PM junctions and vinculin. (**Aa**) PANC-1 cells transfected with YFP–STIM1 were treated with CPA then fixed with PFA and immunostained for vinculin. The scale bar represents 10 μm. (**Ab**) Distances between vinculin-stained focal adhesions and the nearest STIM1 puncta. The histogram is based on 84 measurements taken from seven cells. (**Ac**) High magnification images of closely located vinculin (red) and STIM1 puncta (green). The scale bars represent 1 μm. (**B**) The relative distribution of linker-delineated junctions and vinculin in PANC-1 cells. In these experiments ER Ca^2+^ stores have not been depleted. PANC-1 cells transfected with rapamycin-inducible linkers were treated with rapamycin to reveal the ER–PM junctions, then fixed with PFA and immunostained for vinculin. The green colour in the left-hand panel highlights the structures in which staining of ER and PM markers is co-localized following the addition of rapamycin. Vinculin is shown in red. The scale bar represents 10 μm.

**Figure 5:**
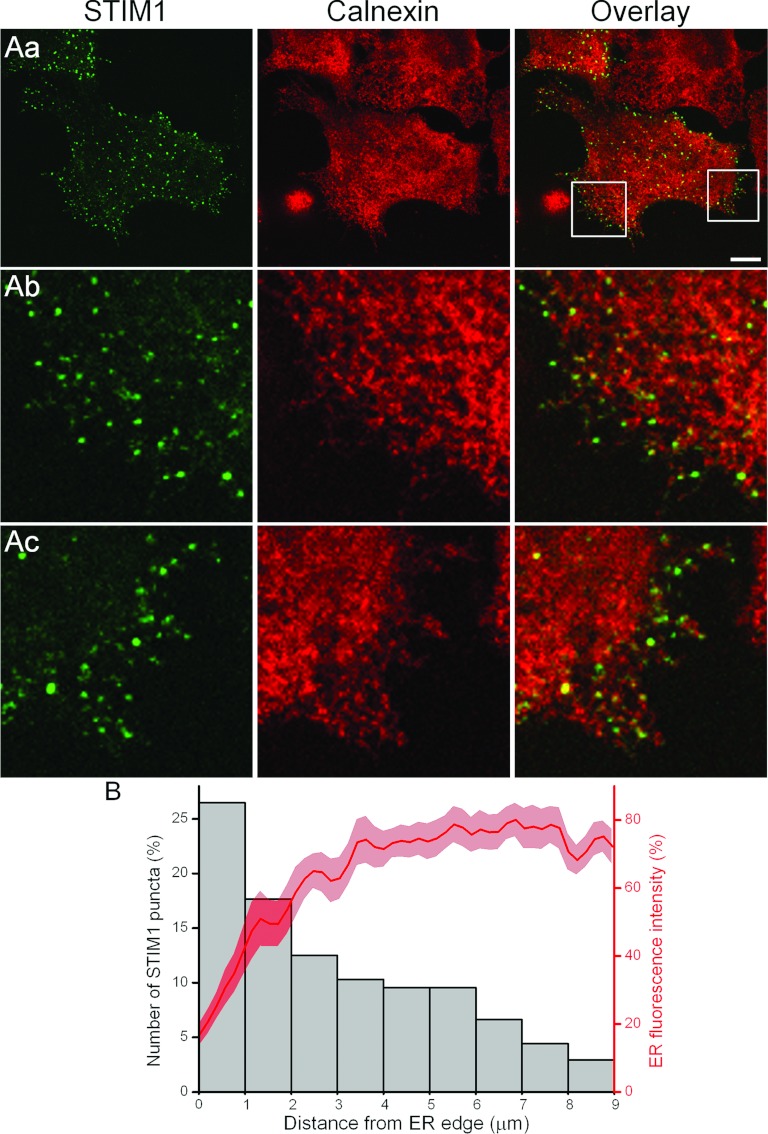
ER–PM junctions and the distribution of ER strands (**A**) Relationship between STIM1 puncta (green) and calnexin (red). YFP–STIM1-transfected PANC-1 cells were treated with CPA in full medium with reduced Ca^2+^, then fixed with PFA and immunostained for calnexin. (**Aa**) The relative positioning of STIM1 puncta and calnexin. The boxes in (**Aa**) highlight regions with differently shaped ER protrusions shown as expanded fragments in (**Ab**) and (**Ac**). The scale bar represents 10 μm. (**Ab**) is a fragment of (**Aa**) [corresponds to the left-hand box in (**Aa**)]. (**Ac**) is another fragment of (**Aa**) [corresponds to the right-hand box in (**Aa**)]. Note the decrease in ER density at the periphery (the intensity of calnexin staining decreases and the staining separates into individual strands) and the presence of STIM1 puncta in the peripheral regions (**Ab** and **Ac**). (**B**) The ER density and the number of STIM1 puncta in each interval measured from the edge of the ER are shown on the same histogram. The histogram is based on 136 measurements of puncta collected from eight cells. For each cell 3.6 μm of a randomly selected outline of the edge of calnexin staining was selected and the number of puncta belonging to the depicted distance intervals from the edge (up to 9 μm from the edge) were counted, expressed as the percentage of the total number of puncta and displayed against the appropriate intervals. The fluorescence of immunolabelled calnexin was measured at the specified distances (up to 9 μm) from the edge and displayed as an average trace±S.E.M. Measurements of calnexin fluorescence and STIM1 puncta were conducted in the same cells and the same regions.

We next characterized the positioning of ER–PM junctions with respect to the integral elements of migratory cells. Using simultaneous labelling of STIM1 and actin we found that the peripheral group of ER–PM junctions is located in close proximity to the inner layer of polymerized actin (Figure 3A). The majority of junctions (STIM1 puncta) of this group were found closer than 0.5 μm (*n*=99 measurements and *n*=9 cells) to the nearest strands of polymerized actin. The ER–PM junctions revealed using ER–PM linkers were also found in close proximity to actin (*n*=27, Figure 3B). Simultaneous staining for vinculin and STIM1 revealed the relative positioning of focal adhesions and ER–PM junctions (Figure 4Aa). We observed that ER–PM junctions can be found in close proximity to focal adhesions (Figures 4Aa–Ac). Indeed the vast majority of focal adhesions have at least one STIM1-decorated ER–PM junction within 0.5 μm of its border (*n*=84 measurements and *n*=7 cells, Figure 4Ab); the reverse is not necessarily the case since there are more ER–PM junctions than focal adhesions. The peripheral ER–PM junctions, revealed by ER–PM linkers, can also be found in close proximity to focal adhesions (*n*=16, Figure 4B). One of the possible reasons for the local clustering of ER–PM junctions is an increased density of actual ER. We therefore compared the distribution of ER and ER–PM junctions. The simultaneous staining of calnexin and STIM1 puncta (*n*=21) revealed the relative positioning of ER strands and ER–PM junctions. We found that the density of ER was not increased, but declined at the cell periphery (Figure 5). Junctions were not found in the cell regions devoid of ER, but, interestingly, peripheral junctions concentrated specifically in the regions with reduced ER density (Figures 5Ab, 5Ac and 5B). This correlates well with the reported area of preferential local Ca^2+^ signalling near the leading edge of migrating cells which has a relatively low ER density [15].

The results of the present study are in agreement with the previously published studies describing localized Ca^2+^ signalling events at the leading edge of migrating cells [14,15]. Indeed the ER–PM junctions, continuously forming in the proximity of the leading edge of migrating cells, will be ideally positioned to serve as platforms for local SOCE that could refill the Ca^2+^-releasing stores and possibly produce their own local Ca^2+^ gradients. Furthermore, the close proximity of the ER–PM junctions to the inner edge of actin and focal adhesions suggests that these structures which are crucial for migration could be particularly sensitive to the signalling events (e.g. Ca^2+^ and/or cAMP signalling) that develop in the junctions.

## Online data

Supplementary data

Supplementary Movie S1. Dynamics of ER--PM junctions in a store depleted migrating cell

Supplementary Movie S2. Dynamics of ER--PM junctions during tail retraction in a store depleted migrating cell
