# Supplementary material for: Saltatory formation, sliding and dissolution of ER–PM junctions in migrating cancer cells
Source: Biochem J. 2013 Mar 14;451(Pt 1):25–32. doi: 10.1042/BJ20121864 (PMC3632088; doi:10.1042/BJ20121864)
Supplement: Supplementary data [file bj4510025add.pdf]

BJ Cell

Biochem. J. (2013) **451**, 25–32 (Printed in Great Britain) doi:10.1042/BJ20121864

online data multimedia

## SUPPLEMENTARY ONLINE DATA

# Saltatory formation, sliding and dissolution of ER–PM junctions in migrating cancer cells

Hayley DINGSDALE*, Emmanuel OKEKE*, Muhammad AWAIS†, Lee HAYNES*, David N. CRIDDLE*, Robert SUTTON† and Alexei V. TEPIKIN*[1]

*Department of Cellular and Molecular Physiology, The University of Liverpool, Crown Street, Liverpool L69 3BX, U.K., and †NIHR (National Institute of Health Research) Liverpool Pancreas Biomedical Research Unit, The University of Liverpool, Crown Street, Liverpool L69 3BX, U.K.

Biochemical Journal www.biochemj.org

## MATERIALS AND METHODS

### Migration assay

Cells were seeded in 35-mm high-$\mu$ dishes with single culture inserts (Ibidi) and maintained at 5 % $CO_2$ and 95 % humidity. The insert was removed after the cells reached confluency (48 h) and the appropriate treatment was initiated. The dishes were imaged at 0 and 48 h after removal of the insert and the start of the treatment. The difference in cell-covered area after 48 h was analysed using WimScratch software. During migration the cells were kept in DMEM (the basal medium containing 0 mM $Ca^{2+}$ to which $CaCl_2$ was added to attain the required $Ca^{2+}$ concentration of 1 mM $Ca^{2+}$) and supplemented with 10 % (v/v) FBS, 100 units/ml penicillin, 100 $\mu$g/ml streptomycin and 292 $\mu$g/ml glutamine. CPA (30 $\mu$M) was added to this solution specifically for the experiments investigating the effect of $[Ca^{2+}]_{ER}$ depletion on cell migration. In the control experiments and experiments involving CPA treatment the temperature of solution was maintained at 37 °C. To test the effect of increased temperature on the cell migration the temperature was increased to 40 °C.

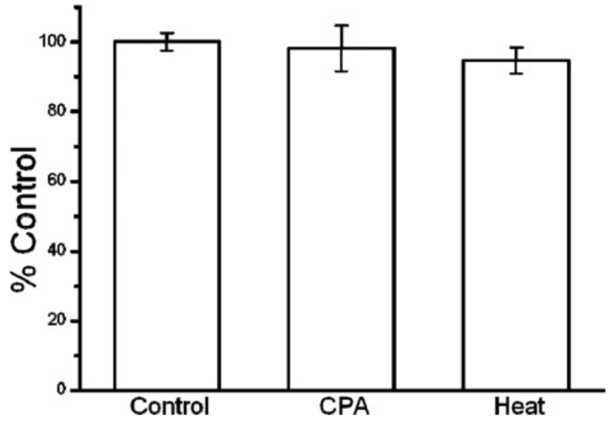


**Figure S1 Neither store depletion nor heat treatment prevents PANC-1 migration**

PANC-1 cells were assessed for their ability to migrate in different conditions using the migration assay described in the Materials and methods section. The change in cell-covered area was normalized to that of the average of the controls from the same experimental set. Results are means S.E.M., $n = 13$ (control), 3 (CPA) and 5 (heat-treated).

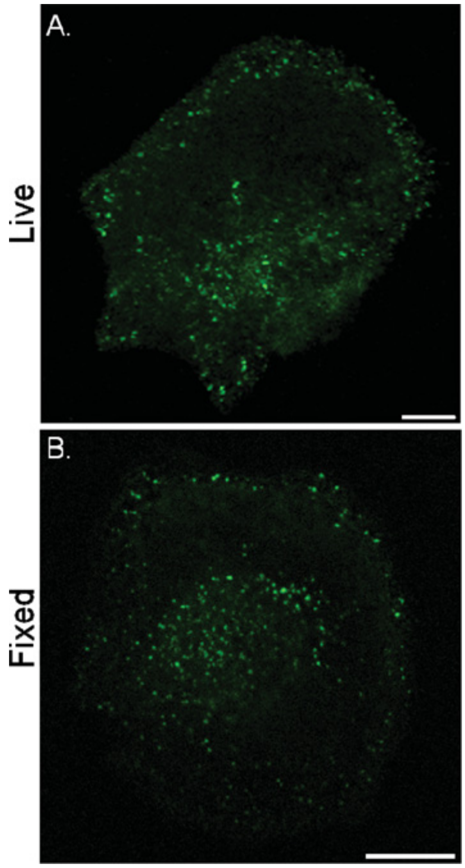


**Figure S2 Peripheral ER-PM junctions revealed by a YFP-labelled STIM1 EF-hand mutant (D76A)**

(**A**) A live PANC-1 cell expressing YFP–STIM1 (D76A). In these experiments ER $Ca^{2+}$ stores have not been depleted. Cells were imaged using an inverted confocal microscope. The confocal section closest to the coverslip is shown on this and other Supplementary Figures. (**B**) A fixed PANC-1 cell expressing YFP–STIM1 (D76A). Cells were fixed with PFA. The conditions for incubation before fixation were the same for (**A**) and (**B**). Scale bars, 10 $\mu$m.

[1] To whom correspondence should be addressed (email a.tepikin@liv.ac.uk).

© 2013 The Author(s)
The author(s) has paid for this article to be freely available under the terms of the Creative Commons Attribution Licence (CC-BY)(http://creativecommons.org/licenses/by/3.0/) which permits unrestricted use, distribution and reproduction in any medium, provided the original work is properly cited.

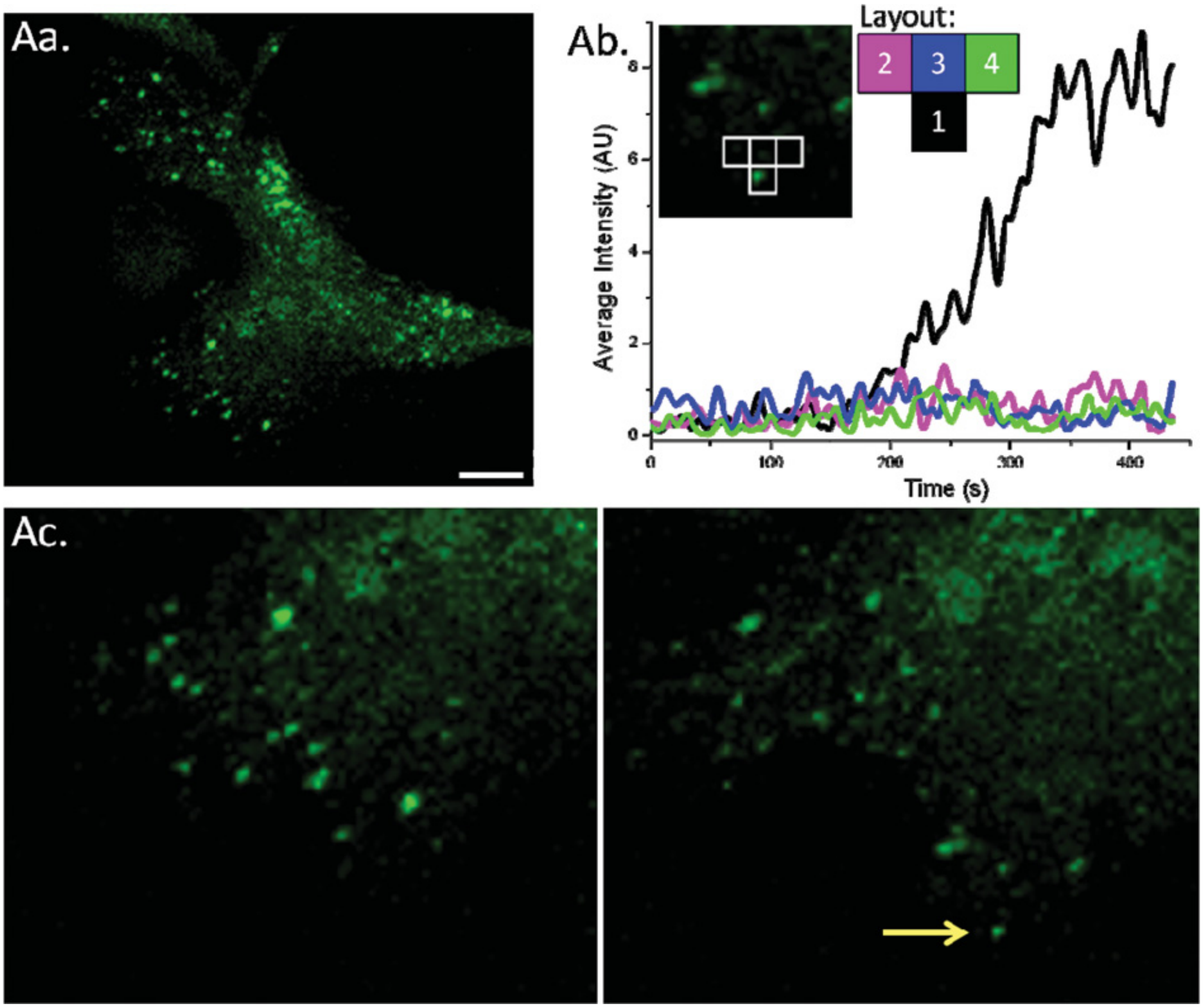


**Figure S3 Saltatory formation of ER–PM junctions revealed using temperature-induced STIM1 puncta**

In these experiments ER $Ca^{2+}$ stores have not been depleted. YFP–STIM1-transfected PANC-1 cells were imaged at 40°C using a confocal microscope. (**Aa**) Image of a cell containing the area selected for analysis of puncta formation. The scale bar represents 10 $\mu$m. (**Ab**) Fragment of (**Aa**), with four regions of interest, one includes the newly formed punctum (region 1) and three (regions 2–4) include the neighbouring peripheral regions of the cell. The graph shows fluorescence intensity over time in each region;the colour of the traces corresponds to the colour of the regions according to the depicted layout. The increase in fluorescence in region 1 reflects the punctum formation. (**Ac**) Images showing the cell region before (left-hand panel) and after (right-hand panel) the punctum appearance. The yellow arrow shows the punctum of interest [the same as in (**Ab**)]. AU, arbitrary units.

© 2013 The Author(s)
The author(s) has paid for this article to be freely available under the terms of the Creative Commons Attribution Licence (CC-BY)(http://creativecommons.org/licenses/by/3.0/) which permits unrestricted use, distribution and reproduction in any medium, provided the original work is properly cited.

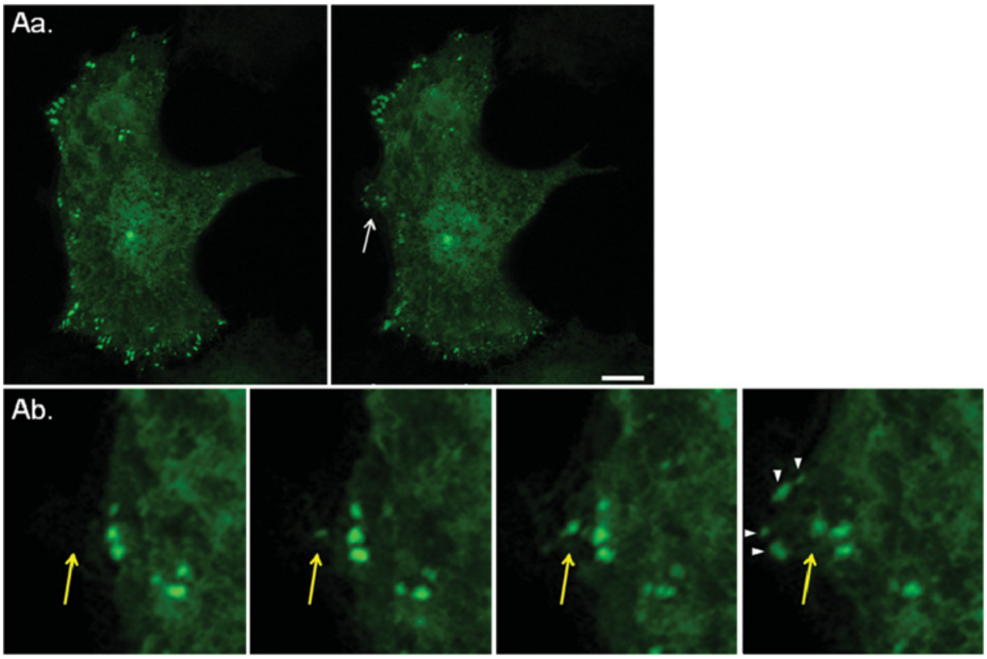


**Figure S4 Saltatory formation of ER–PM junctions can be observed in a cell expressing a STIM1 mutant that cannot bind to EB1 (end-binding protein 1)**

YFP–STIM1(NN)-transfected PANC-1 cells were treated with CPA and imaged using a confocal microscope. (**Aa**) Images of a cell before (left-hand panel) and after (right-hand panel) formation of the puncta of interest. The part of the cell undergoing forward movement accompanied by the formation of puncta (see **Ab**) is shown by an arrow on the right panel. The scale bar represents 10 $\mu$m. (**Ab**) Images showing cell region before, during and after punctum appearance, with yellow arrows highlighting the newly formed punctum. Note four other puncta that suddenly appeared when the cell moves further forward (white arrowheads).

© 2013 The Author(s)
The author(s) has paid for this article to be freely available under the terms of the Creative Commons Attribution Licence (CC-BY)(http://creativecommons.org/licenses/by/3.0/) which permits unrestricted use, distribution and reproduction in any medium, provided the original work is properly cited.

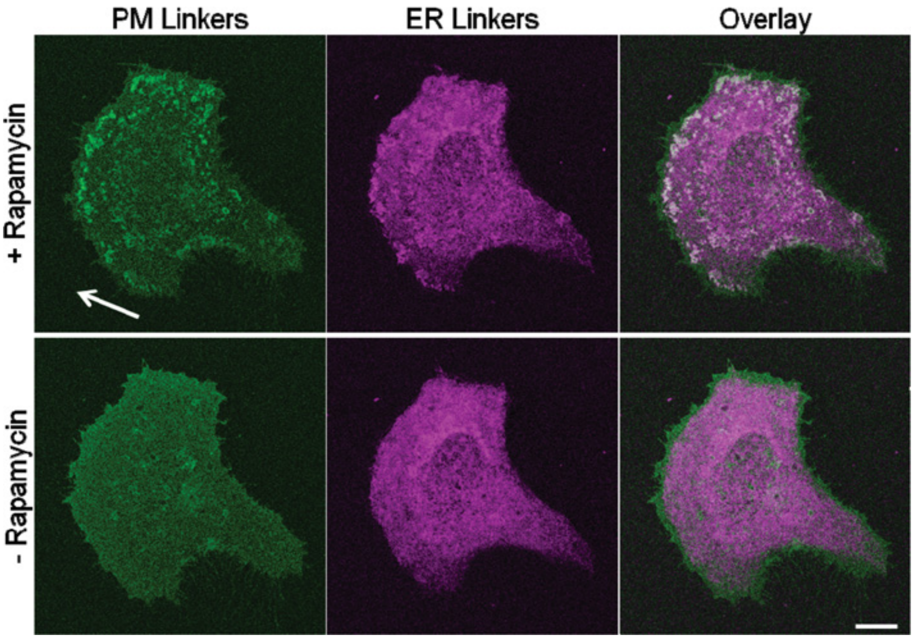


**Figure S5 Rapamycin treatment triggers co-clustering of ER and PM linkers revealing the localization of the ER–PM junctions**

The Figure shows the same cell as in Figure 2 of the main text. The lower panels show the distribution of fluorescence before the application of rapamycin. The upper panels show the distribution of fluorescence after the application of rapamycin (as in Figure 2 of the main text) the increased co-localization of the two constructs (white staining on overlay image) reveals the location of the ER–PM junctions. The cells were transfected with PM-targeted LL–FKBP–mRFP and ER-targeted CFP–FRB–LL and imaged live. The arrow shows direction of migration. The scale bar represents 10 $\mu$m.

Received 13 December 2012/16 January 2013
Published as BJ Immediate Publication 16 January 2013, doi:10.1042/BJ20121864

© 2013 The Author(s)
The author(s) has paid for this article to be freely available under the terms of the Creative Commons Attribution Licence (CC-BY)(http://creativecommons.org/licenses/by/3.0/) which permits unrestricted use, distribution and reproduction in any medium, provided the original work is properly cited.
